# Cannabidiol Mitigates Pollution-Induced Inflammatory, Oxidative, and Barrier Damage in Ex Vivo Human Skin

**DOI:** 10.3390/biom16010010

**Published:** 2025-12-20

**Authors:** Wannita Klinngam, Orathai Loruthai, Sornkanok Vimolmangkang

**Affiliations:** 1 National Nanotechnology Center (NANOTEC), National Science and Technology Development Agency, Pathum Thani 12120, Thailand; orathai@nanotec.or.th; 2 Department of Pharmacognosy and Pharmaceutical Botany, Faculty of Pharmaceutical Sciences, Chulalongkorn University, Bangkok 10330, Thailand; sornkanok.v@pharm.chula.ac.th; 3 Research Cluster for Cannabis and Its Natural Substances, Faculty of Dentistry, Chulalongkorn University, Bangkok 10330, Thailand

**Keywords:** cannabidiol (CBD), ex vivo skin model, particulate matter (PM), skin inflammation, oxidative stress, skin structural integrity

## Abstract

Airborne particulate matter (PM) is a major environmental pollutant that accelerates skin aging, inflammation, and barrier impairment. Cannabidiol (CBD), a non-psychoactive phytocannabinoid derived from *Cannabis sativa*, has shown anti-inflammatory and cytoprotective effects, yet its role in protecting full-thickness human skin from pollution-induced damage remains unclear. In this study, human full-thickness ex vivo skin explants were topically exposed to PM (0.54 mg/cm^2^) and treated with CBD (6.4 mM) administered via the culture medium for 48 h. Proinflammatory mediators (interleukin-6, IL-6; matrix metalloproteinase-1, MMP-1; cyclooxygenase-2, COX-2), oxidative stress markers (reactive oxygen species, ROS; 8-hydroxy-2′-deoxyguanosine, 8-OHdG), the xenobiotic sensor aryl hydrocarbon receptor (AhR), extracellular matrix proteins (procollagen type I C-peptide, PIP; fibrillin), and the barrier protein filaggrin were quantified using ELISA and immunofluorescence. PM exposure triggered significant inflammation, oxidative stress, AhR induction, extracellular matrix degradation, and barrier disruption. CBD selectively counteracted these effects by reducing IL-6, MMP-1, COX-2, ROS, and 8-OHdG levels, downregulating AhR expression, and restoring PIP, fibrillin, and filaggrin expression. No measurable effects were observed in unstressed control tissues. These results demonstrate that CBD protects human skin from PM-induced molecular damage and supports its potential as a functional bioactive ingredient for anti-pollution applications.

## 1. Introduction

Air pollution remains one of the most pressing global environmental threats, particularly in densely populated urban areas. As the body’s outermost barrier, the skin is continuously exposed to airborne pollutants such as particulate matter (PM), polycyclic aromatic hydrocarbons (PAHs), and heavy metals [1,2]. These pollutants can penetrate the skin barrier and accumulate in deeper layers, triggering oxidative stress, inflammation, and premature aging [3,4,5]. Mechanistically, pollutant exposure initiates a cascade of responses, including excessive production of reactive oxygen species (ROS), depletion of endogenous antioxidants, activation of proinflammatory signaling, and induction of the aryl hydrocarbon receptor (AhR) [5,6,7,8]. These pathways collectively impair skin integrity by degrading extracellular matrix (ECM) components and barrier proteins such as collagen and filaggrin [9,10].

While topical skincare products containing antioxidants and anti-inflammatory agents are widely used, many offer only superficial protection and are insufficient against ultrafine pollutants, especially PM2.5, which can penetrate the stratum corneum and reach deeper skin layers or systemic circulation [11,12,13]. Nutricosmetics, which are oral or systemic supplements that support skin health from within, have therefore gained interest. Products such as Zeropollution^®^ have shown efficacy in preclinical and clinical models, highlighting the need for actives with systemic action [13,14]. However, few compounds have been validated in biologically relevant human skin models that mimic real-world exposure. Most anti-pollution studies rely on two-dimensional monocultures, which lack the structural and cellular complexity of human skin [15,16,17]. In contrast, full-thickness ex vivo human skin retains stratified epidermal and dermal layers, extracellular matrix architecture, resident immune cells, and an intact barrier, making it a physiologically relevant model for preclinical evaluation, particularly for nutricosmetic applications [18].

Cannabidiol (CBD), a non-psychoactive phytocannabinoid from *Cannabis sativa*, has shown antioxidant, anti-inflammatory, and barrier-supporting effects [19,20]. Unlike tetrahydrocannabinol (THC), CBD does not produce psychoactive effects and has been evaluated by international regulatory bodies as having no evidence of abuse or dependence potential in humans [21,22]. Mechanistically, CBD modulates pathways involved in skin homeostasis, including nuclear factor erythroid 2–related factor 2 (NRF2), cyclooxygenase-2 (COX-2), and cytokines such as interleukin-6 (IL-6) and tumor necrosis factor-alpha (TNF-α) [23,24,25,26,27,28,29]. In keratinocyte monocultures and reconstructed epidermis, CBD mitigates UVB- and ROS-induced oxidative damage while promoting epidermal differentiation and barrier proteins such as filaggrin [26,27,28,30]. A previous study has shown that CBD reduced IL-6 and IL-1α in keratinocytes exposed to PM, while exerting limited effects on extracellular matrix markers in basal ex vivo skin models [22]. However, its efficacy in pollution-challenged human skin, particularly in full-thickness ex vivo models and in the context of systemic-like exposure, remains underexplored.

In this study, we used a full-thickness human ex vivo skin model to assess the anti-pollution effects of CBD delivered via the culture medium, simulating systemic exposure. Medium-based delivery was chosen because full-thickness ex vivo skin lacks vascular perfusion, making administration through the medium the only feasible way to mimic circulating bioactives reaching the dermis, which is relevant for nutricosmetic or orally delivered CBD [31,32]. This approach also avoids variability from topical penetration and formulation effects, enabling controlled evaluation of CBD activity and addressing the current gap in which most anti-pollution and CBD–skin studies focus solely on topical models. Skin explants were then topically challenged with standardized Urban Dust (SRM 1649b), a certified reference material widely used as a representative urban particulate mixture containing a fine (PM2.5-inclusive) fraction and associated organic and inorganic pollutants, and CBD’s effects were evaluated under both stressed and unstressed conditions. Biomarkers assessed included inflammatory mediators (IL-6, MMP-1, COX-2), oxidative stress markers (ROS, 8-OHdG), the environmental sensor AhR, ECM proteins (PIP, fibrillin), and the barrier protein filaggrin. Ascorbic acid (vitamin C) served as a reference antioxidant and anti-pollution control [33]. Taken together, this study investigates whether CBD can attenuate PM-induced inflammation, oxidative stress, and loss of extracellular matrix and barrier proteins in full-thickness human ex vivo skin using a physiologically relevant preclinical model.

## 2. Materials and Methods

### 2.1. Human Ex Vivo Skin Model Preparation

Full-thickness abdominal skin samples were obtained from five healthy female donors (aged 36–63 years) undergoing elective abdominoplasty, following approval by the Institutional Ethics Committee of Yanhee International Hospital, Thailand (Approval No. FM-YGH-024, approved 8 June 2023). Written informed consent was obtained from all participants prior to tissue collection, and all procedures were conducted in accordance with the Declaration of Helsinki.

Skin preparation and culture were performed as previously described, with minor modifications [33]. Briefly, subcutaneous adipose tissue was carefully removed, and skin samples were disinfected and rinsed in phosphate-buffered saline (PBS; Gibco, Waltham, MA, USA) containing Amphotericin B (Millipore Sigma, Burlington, MA, USA). Circular full-thickness skin explants (6 mm diameter) were placed onto collagen/Matrigel^®^ (Corning, Tewksbury, MA, USA)-coated transwell inserts and cultured overnight in Dulbecco’s Modified Eagle Medium (DMEM; Gibco, Waltham, MA, USA) supplemented with 10% fetal bovine serum (FBS; Gibco, Waltham, MA, USA), GlutaMAX™ (Gibco, Waltham, MA, USA), insulin (Millipore Sigma, Burlington, MA, USA), and Primocin (InvivoGen, San diego, CA, USA) prior to experimental treatment.

### 2.2. PM Dose Optimization Study

A preliminary dose–response study was conducted to identify the optimal PM concentration for inducing measurable skin damage in the ex vivo model. Full-thickness skin explants from a single 53-year-old female donor were topically exposed to standardized Urban Dust (SRM 1649b; National Institute of Standards and Technology, Gaithersburg, MD, USA), a certified reference material representing ambient urban PM, at surface doses of 0.25, 0.36, 0.54, and 0.71 mg/cm^2^. For each dose, 10 µL of the PM suspension was applied directly to the tissue surface and incubated for 24 h. A negative control group received 0.5% dimethyl sulfoxide (DMSO; Millipore Sigma, Burlington, MA, USA) without PM.

Tissue viability was evaluated using the MTT assay, and culture supernatants were collected to measure IL-6, MMP-1, and ROS levels. Each condition was tested in triplicate (*n* = 3 tissues per group). Data from this dose optimization study was used to determine the PM concentration employed in the main experiment (see Section 2.4). This preliminary dose optimization experiment used a different protocol from the main CBD studies and served only to select the PM dose; it was not included in the main biological analysis, so the characteristics of this single donor do not materially influence the interpretation of the main study conclusions.

### 2.3. CBD Concentration Selection Study

To identify the optimal CBD concentration for downstream analyses, full-thickness skin explants from a 40-year-old female donor were topically exposed to 10 µL of PM (0.54 mg/cm^2^) and simultaneously treated with CBD isolate (batch no. L.0416102301B, Eastern Spectrum Group Co., Ltd., Ratchaburi, Thailand). This CBD batch was analytically verified by high-performance liquid chromatography with photodiode array detection (HPLC-PDA), confirming 99.40% cannabidiol content with non-detectable levels of cannabidiolic acid (CBDA), delta-9-tetrahydrocannabinol (Δ9-THC), and tetrahydrocannabinolic acid (THC-A), all below their respective detection limits (LOD 0.0005% *w*/*w*).

CBD was delivered via the culture medium at final concentrations of 0.95, 3.2, 4.8, and 6.4 mM. Although medium-based administration does not reproduce human systemic pharmacokinetics, it is the only practical way in this ex vivo model to mimic circulating CBD reaching the dermis [31,32]. Control groups included: (i) 0.5% DMSO without PM (vehicle control) and (ii) 0.5% DMSO with PM. Explants were cultured for 48 h in supplemented DMEM, with daily replacement of medium and treatments. Supernatants were collected at 24 and 48 h for quantification of MMP-1 and IL-6, respectively. At 48 h, tissues were harvested for MTT viability assay and immunofluorescence analysis of COX-2 and collagen type I. Each condition was tested in triplicate (*n* = 3 tissues per group). These findings informed the CBD dose selection in Section 2.4. This concentration-selection step was not part of the biological analysis, so donor age does not materially influence the interpretation of the study’s conclusions.

### 2.4. Assessment of Anti-Pollution Effects of CBD

The protective effects of CBD against pollution-induced skin damage were evaluated using full-thickness human ex vivo skin explants from three female donors (aged 36–63 years), assigned to eight treatment groups. Four groups were maintained under non-polluted conditions: (i) water control (negative control for ascorbic acid), (ii) ascorbic acid (250 µM; Millipore Sigma, Burlington, MA, USA), (iii) 0.5% DMSO (vehicle control for CBD; this concentration is non-cytotoxic in skin fibroblasts and is expected to have only minimal effects on penetration under our medium-based exposure conditions) [34], and (iv) CBD (6.4 mM). The remaining four groups were topically exposed to PM (10 µL, 0.54 mg/cm^2^) and treated identically via the culture medium: (v) PM + water, (vi) PM + ascorbic acid, (vii) PM + 0.5% DMSO, and (viii) PM + CBD. 250 µM ascorbic acid was selected based on previous skin-related oxidative stress studies and our ex vivo data showing that this concentration is commonly used, effective, and non-cytotoxic, while avoiding potential pro-oxidant effects observed at higher doses [33,35]. 6.4 mM CBD was selected based on our concentration–response experiment (Section 2.3), where 6.4 mM most effectively reduced PM-induced inflammatory markers without affecting tissue viability. All treatments were applied through the medium to achieve the specified final concentrations.

Explants were cultured for 48 h in supplemented DMEM with daily replacement of treatment medium, followed by a 7 h recovery period in fibroblast serum-free medium (ATCC, Manassas, VA, USA) without PM or test compounds. Post-treatment, MTT assay was used to assess tissue viability. Culture supernatants were collected for analysis of IL-6, MMP-1, ROS, 8-OHdG, and PIP. Fixed tissues were processed for immunofluorescence staining of COX-2, filaggrin, fibrillin, and AhR. Sample sizes were *n* = 9 tissues per group (3 tissues per donor from 3 donors) for biochemical assays and *n* = 6 tissues per group (3 tissues from 2 donors) for immunofluorescence analysis.

### 2.5. Tissue Viability Assessment Using MTT Assay

Tissue viability was assessed using the MTT assay (catalog #M5655; Millipore Sigma, USA), following previously published protocols with minor modifications [33]. Briefly, skin explants were incubated with MTT solution (1 mg/mL) at 37 °C in a humidified 5% CO_2_ atmosphere for 3 h. Following incubation, formazan crystals were solubilized by transferring tissues to isopropanol (catalog #34863; Millipore Sigma, USA) and shaking at 120 rpm for 2 h. Absorbance was measured at 570 nm using a microplate reader (SpectraMax iD5; Molecular Devices, San Jose, CA, USA). Viability was normalized to tissue weight (mg) and expressed as a percentage relative to the non-PM water control. A positive control group was included by treating explants with 1% Triton X-100 (Millipore Sigma, USA) for 24 h to induce complete cell death and confirm assay responsiveness.

### 2.6. Quantification of IL-6, MMP-1, ROS, 8-OHdG, and PIP in Culture Medium

Culture supernatants were analyzed for IL-6 and MMP-1 using AlphaLISA kits (IL-6: #AL353C; MMP-1: #AL242C; Revvity, Waltham, MA, USA), according to the manufacturer’s protocols. Luminescence was detected using an EnVision-Alpha plate reader (Revvity, USA). ROS were quantified using the DCF ROS/RNS Assay Kit (#ab238535; Abcam, Cambridge, MA, USA). Oxidative DNA damage marker 8-OHdG and PIP were measured by ELISA using kits #ab201734 and #ab272198 (Abcam, USA), respectively. Fluorescence and absorbance signals were recorded on a SpectraMax iD5 microplate reader (Molecular Devices, USA).

All analyte values were normalized to total protein content using the Pierce™ 660 nm Protein Assay (#22662; Thermo Fisher Scientific, Waltham, MA, USA) and expressed as a percentage relative to the non-PM vehicle control. Assays were performed in duplicate using media from nine skin explants per group (*n* = 9; 3 tissues per donor from 3 donors).

### 2.7. Immunofluorescence Staining of COX-2, AhR, Fibrillin, Filaggrin, and Collagen Type I

Skin tissues were fixed in 4% paraformaldehyde (#12606S; Cell Signaling Technology, Danvers, MA, USA) and 4% sucrose (#S0389; Millipore Sigma, USA) in PBS, followed by cryoprotection in 30% sucrose. Samples were embedded in Tissue-Tek O.C.T. Compound (Sakura Finetek, Torrance, CA, USA) and sectioned using a cryostat microtome (Leica Biosystems, Deer Park, IL, USA). Sections were washed with PBST (PBS + 0.1% Tween 20; #17606531, Bio-Rad Laboratories, Hercules, CA, USA), quenched with 100 mM ammonium chloride (#213330; Sigma-Aldrich, Burlington, MA, USA) for 10 min, and treated with 0.1% Sudan Black B (#199664; Sigma-Aldrich, Burlington, MA, USA) in 70% ethanol to reduce autofluorescence.

Tissues were permeabilized with 0.1% Triton X-100 and blocked with 1% bovine serum albumin (#A9647; Sigma-Aldrich, USA) for 1 h at room temperature. Sections were incubated overnight at 4 °C with primary antibodies against COX-2 (#ab179800), AhR (#ab190797), fibrillin (#MA512770), filaggrin (#ab81468), and collagen type I (#ab34710) (all from Abcam, Cambridge, UK or Thermo Fisher Scientific, Waltham, MA, USA, as specified). After washing, secondary antibodies were applied: Alexa Fluor 488 goat anti-rabbit IgG, Alexa Fluor 488 goat anti-mouse IgG (Invitrogen, Carlsbad, CA, USA), and DyLight^®^ 594 goat anti-rabbit IgG (#ab96901; Abcam, UK). Sections were rinsed with 300 mM NaCl (#S9888; Sigma-Aldrich, USA) and counterstained with DAPI (Abcam, UK) for 15 min.

Slides were mounted with ProLong™ Gold Antifade Mountant (#9071S; Cell Signaling Technology, Danvers, MA, USA) and imaged at 400× magnification using a Leica TCS SP8 STED confocal microscope (Leica Microsystems, Wetzlar, Germany). For quantitative analysis, one section per sample and five randomly selected fields per section were analyzed using ImageJ v1.54f (NIH, Bethesda, MD, USA). Fluorescence intensity was calculated as mean signal per area after background subtraction and expressed as a percentage relative to the non-PM vehicle control. Sample size: *n* = 6 tissues per group (3 tissues from each of 2 donors).

### 2.8. Statistical Analysis

All data were analyzed using GraphPad Prism version 7 (GraphPad Software, San Diego, CA, USA) and are presented as mean ± standard deviation (SD). For the PM dose optimization and CBD concentration selection studies (Figures S1–S3), one-way ANOVA followed by Tukey’s post hoc test was used to compare all treatment groups. For tissue viability (MTT assay), one-way ANOVA followed by Dunnett’s post hoc test was applied to compare each group against the non-PM vehicle control.

For evaluation of CBD efficacy across the eight experimental groups, one-way ANOVA followed by Bonferroni’s multiple comparisons test was applied to compare selected treatment pairs of interest, rather than all possible pairwise combinations. Statistical significance was defined as *p* < 0.05, with *p*-values adjusted using the Bonferroni correction for multiple comparisons.

## 3. Results

### 3.1. Establishment of a PM-Induced Skin Damage Model

To mimic pollution-induced skin damage, human full-thickness ex vivo skin explants were topically exposed to PM at concentrations of 0.25, 0.36, 0.54, and 0.71 mg/cm^2^ for 24 h. Tissue viability was assessed by MTT assay, alongside quantification of key inflammatory and oxidative stress markers, including IL-6, MMP-1, and ROS. Among the tested doses, 0.54 mg/cm^2^ significantly increased IL-6, MMP-1, and ROS levels relative to untreated controls, without compromising tissue viability (Figure S1). This dose elicited a robust biochemical response in the absence of cytotoxicity and was thus selected for all subsequent experiments evaluating the protective effects of CBD. This preliminary single-donor 24 h pilot was used solely to select 0.54 mg/cm^2^, as a non-cytotoxic, biologically active PM dose and is not intended for direct quantitative comparison with the multi-donor 48 h anti-pollution experiments.

### 3.2. Optimization of CBD Concentration

To determine the optimal concentration of CBD, human ex vivo skin explants were co-treated with PM (0.54 mg/cm^2^) and escalating CBD doses (0.95–6.4 mM) for 48 h. Tissue viability was evaluated via MTT assay, while inflammatory markers (IL-6, MMP-1, COX-2) and collagen type I expression were assessed. PM exposure significantly elevated IL-6, MMP-1, and COX-2 levels, confirming an inflammatory response. Among all concentrations tested, 6.4 mM CBD most effectively and consistently reduced these markers without compromising tissue viability (Figure S2). Furthermore, collagen type I expression, which was suppressed by PM exposure, was restored by 6.4 mM CBD to levels comparable to untreated control (Figure S3). Based on these outcomes, 6.4 mM was selected as the optimal CBD dose for subsequent experiments due to its potent anti-inflammatory and collagen-preserving effects without cytotoxicity. In the context of this static explant system, this concentration is intended to model maximal local tissue exposure rather than clinically achievable plasma levels.

### 3.3. CBD Suppresses PM-Induced IL-6 and MMP-1 Expression in Human Skin Explants

To assess CBD’s anti-inflammatory effects under pollution stress, human ex vivo skin explants were divided into eight groups. Four groups remained unexposed to PM and received one of the following treatments via the culture medium: water (negative control for ascorbic acid), 250 µM ascorbic acid (positive control), 0.5% DMSO (vehicle control for CBD), or 6.4 mM CBD. The remaining four groups were topically exposed to PM (0.54 mg/cm^2^) and received the same respective treatments. This design allowed direct comparison between basal and pollutant-challenged conditions.

**Figure 1:**
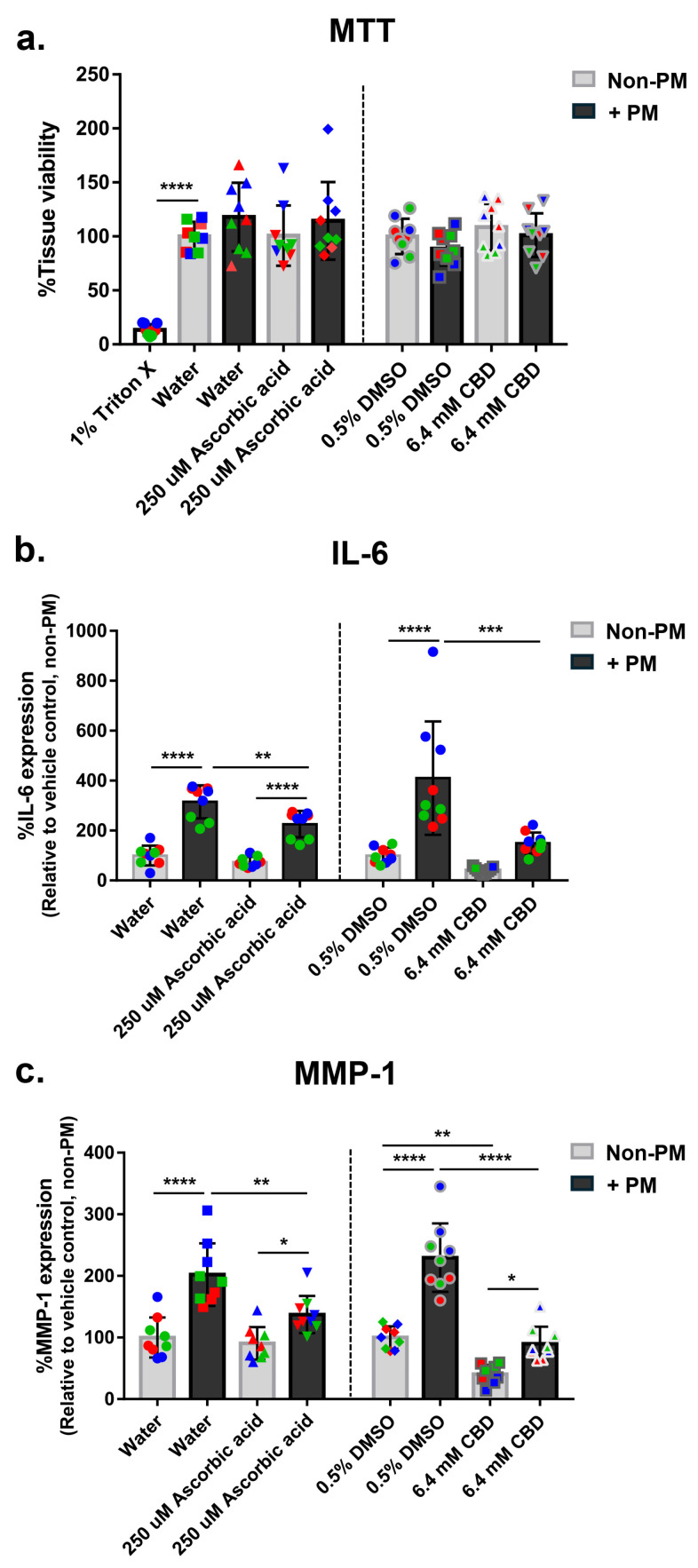
CBD attenuates PM-induced inflammation and matrix degradation in human ex vivo skin explants without compromising tissue viability. Full-thickness human skin explants from three donors (ages 36–63 years; *n* = 9 tissues per group; 3 tissues per donor) were treated for 48 h with 6.4 mM CBD, 250 µM ascorbic acid, water, or 0.5% DMSO, with or without topical PM exposure (0.54 mg/cm^2^). A 1% Triton X-100 control was included for MTT assay validation. (**a**) Tissue viability; (**b**) IL-6 expression; (**c**) MMP-1 expression, shown as % relative to the non-PM vehicle control. Each dot represents an individual explant; dot colors indicate donor origin (Red = donor 1 [59 years], Blue = donor 2 [63 years], Green = donor 3 [36 years]). Gray bars represent non-PM groups; black bars indicate PM-exposed groups. Data are shown as mean ± SD. Asterisks indicate significance: * *p* < 0.05, ** *p* < 0.01, *** *p* < 0.001, **** *p* < 0.0001 (one-way ANOVA with Bonferroni’s post hoc test).

Tissue viability, assessed by MTT assay, confirmed no cytotoxicity in any treatment group (Figure 1a). Only the Triton X-100 positive control reduced viability, validating the assay. Small apparent increases in the water-PM and ascorbic acid-PM groups relative to the non-PM water control were not statistically significant and fell within normal experimental variability. PM exposure significantly increased IL-6 secretion, with a mean 3.4-fold increase across all vehicle and ascorbic acid groups (Figure 1b). Ascorbic acid reduced IL-6 levels by 28% under PM conditions, while CBD reduced IL-6 by 63% compared to the DMSO–PM group. Neither compound affected IL-6 levels under non-PM conditions, suggesting stimulus-specific anti-inflammatory activity (Figure 1b). Similarly, PM exposure increased MMP-1 expression by an average of 2.0-fold across all control and treatment groups (Figure 1c). Ascorbic acid reduced MMP-1 levels by 32% under PM conditions, while CBD significantly reduced MMP-1 expression by 61% under PM stress and 60% in non-PM conditions, indicating broader matrix-preserving effects (Figure 1c). These findings demonstrate that CBD strongly attenuates PM-induced inflammation and ECM degradation without affecting tissue viability, supporting its potential as a protective agent against pollutant-induced skin damage.

### 3.4. CBD Attenuates PM-Induced COX-2 Expression in Human Skin Explants

**Figure 2:**
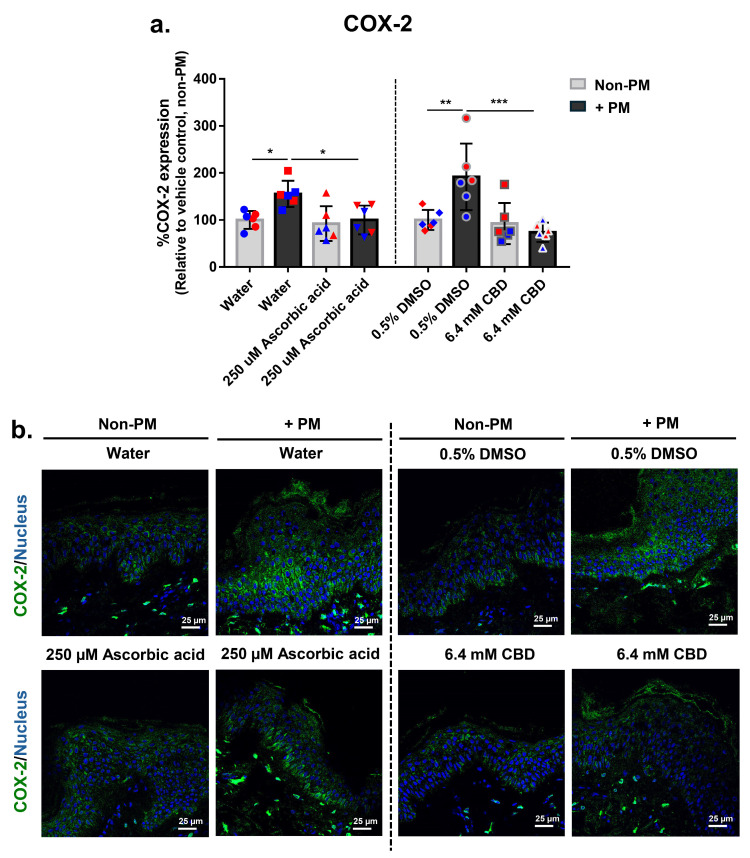
CBD suppresses PM-induced COX-2 expression in human ex vivo skin explants. Full-thickness human skin explants from two donors (ages 59 and 63 years; *n* = 6 tissues per group; 3 tissues per donor) were treated for 48 h with 6.4 mM CBD, 250 µM ascorbic acid, water, or 0.5% DMSO, with or without topical PM exposure (0.54 mg/cm^2^). (**a**) Quantification of COX-2 protein expression in skin tissues by immunofluorescence staining, expressed as % COX-2 expression relative to the non-PM vehicle control; (**b**) Representative immunofluorescence images from donor 1 showing COX-2 (green) and nuclei (blue). Scale bars = 25 µm. Each dot in (**a**) represents an individual explant; dot colors indicate donor origin (Red = donor 1 [59 years], Blue = donor 2 [63 years]). Gray bars represent non-PM groups; black bars indicate PM-exposed groups. Data are shown as mean ± SD. Asterisks indicate significance: * *p* < 0.05, ** *p* < 0.01, *** *p* < 0.001 (one-way ANOVA with Bonferroni’s post hoc test).

To further investigate the anti-inflammatory effects of CBD, COX-2 expression was evaluated via immunofluorescence staining and quantitative image analysis in full-thickness human skin explants. As shown in Figure 2a,b, PM exposure significantly increased COX-2 expression in both vehicle control groups (water and 0.5% DMSO), with levels rising on average 1.7-fold compared to their non-PM counterparts, confirming an inflammatory response.

Under PM conditions, ascorbic acid reduced COX-2 expression by 35%, with no effect observed in non-PM-treated tissues. CBD produced a pronounced effect, reducing PM-induced COX-2 expression by 61% while leaving basal COX-2 levels unchanged (Figure 2a,b). These results suggest that both compounds selectively suppress inducible COX-2 expression. Collectively, these findings further support the anti-inflammatory activity of CBD in PM-exposed human skin, complementing its previously observed effects on IL-6 and MMP-1. Importantly, CBD’s selective suppression under inflammatory conditions indicates its potential to mitigate pollution-induced skin inflammation without disrupting normal skin homeostasis.

### 3.5. CBD Reduces PM-Induced Oxidative Stress in Human Skin Explants

**Figure 3:**
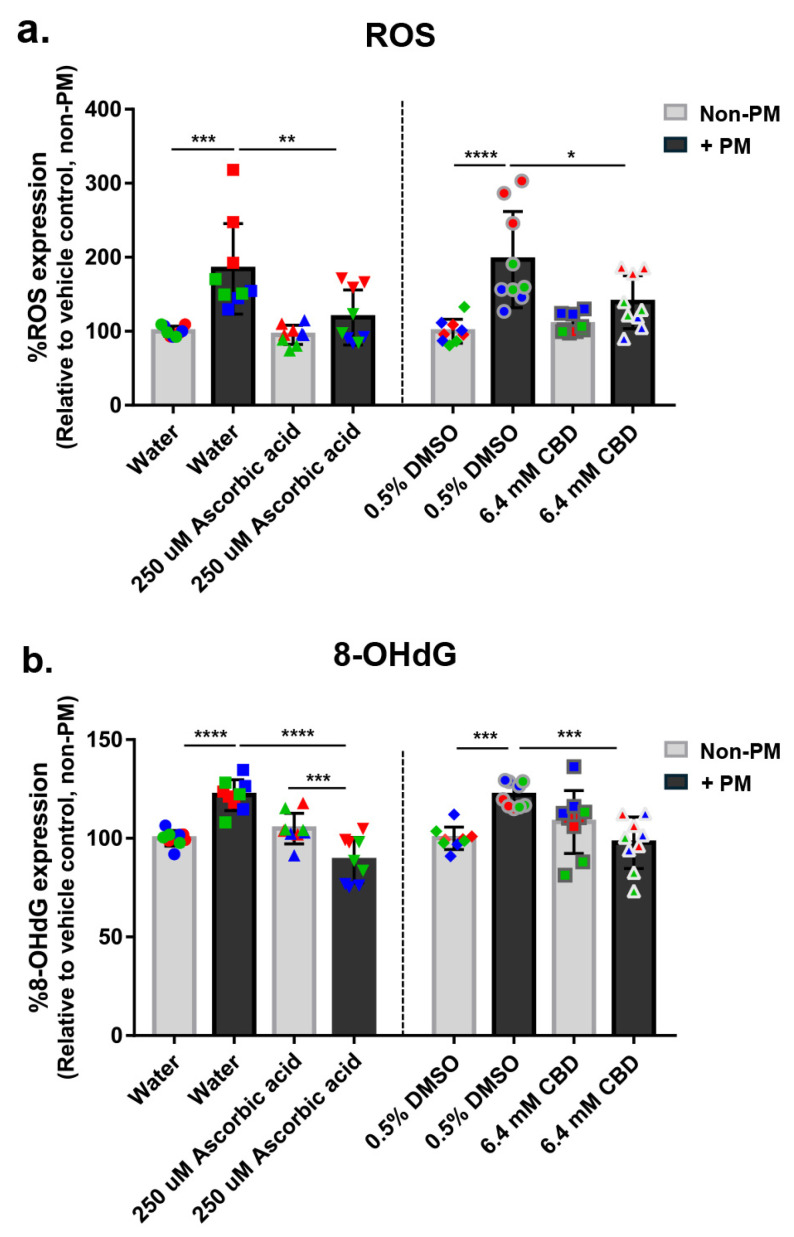
CBD reduces PM-induced oxidative stress in human ex vivo skin explants. Full-thickness human skin explants from three donors (ages 36–63 years; *n* = 9 tissues per group; 3 tissues per donor) were treated for 48 h with 6.4 mM CBD, 250 µM ascorbic acid, water, or 0.5% DMSO, with or without topical PM exposure (0.54 mg/cm^2^). (**a**) ROS level; (**b**) 8-OHdG expression were quantified and reported as % relative to the non-PM vehicle control. Each dot represents an individual explant; dot colors indicate donor origin (Red = donor 1 [59 years], Blue = donor 2 [63 years], Green = donor 3 [36 years]). Gray bars represent non-PM groups; black bars indicate PM-exposed groups. Data are shown as mean ± SD. Asterisks indicate significance: * *p* < 0.05, ** *p* < 0.01, *** *p* < 0.001, **** *p* < 0.0001 (one-way ANOVA with Bonferroni’s post hoc test).

To evaluate the antioxidant effects of CBD, ROS and oxidative DNA damage marker 8-OHdG were measured in human ex vivo skin explants under PM exposure (Figure 3a,b). PM treatment significantly increased ROS levels by an average of 1.9-fold in both vehicle control groups (water and DMSO), confirming oxidative stress induction. Ascorbic acid reduced ROS levels by 36% in PM-exposed tissues, with no effect under non-PM conditions. Similarly, CBD decreased PM-induced ROS by 29%, while baseline ROS levels remained unchanged, indicating pollutant-selective antioxidant activity (Figure 3a).

In line with ROS findings, 8-OHdG levels were elevated by an average of 1.2-fold in PM-treated vehicle controls, reflecting increased oxidative DNA damage (Figure 3b). Ascorbic acid reduced 8-OHdG by 27% under PM exposure, lowering levels below even those of the non-PM ascorbic group. CBD treatment reduced 8-OHdG by 20% in PM-challenged tissues, again with no effect under non-PM conditions (Figure 3b). These data demonstrate that CBD effectively reduces oxidative stress and DNA damage induced by PM, without disturbing redox homeostasis in unstressed skin, highlighting its potential as a redox-modulating agent for environmentally exposed skin.

### 3.6. CBD Attenuates PM-Induced Aryl Hydrocarbon Receptor (AhR) Expression in Human Skin Explants

**Figure 4:**
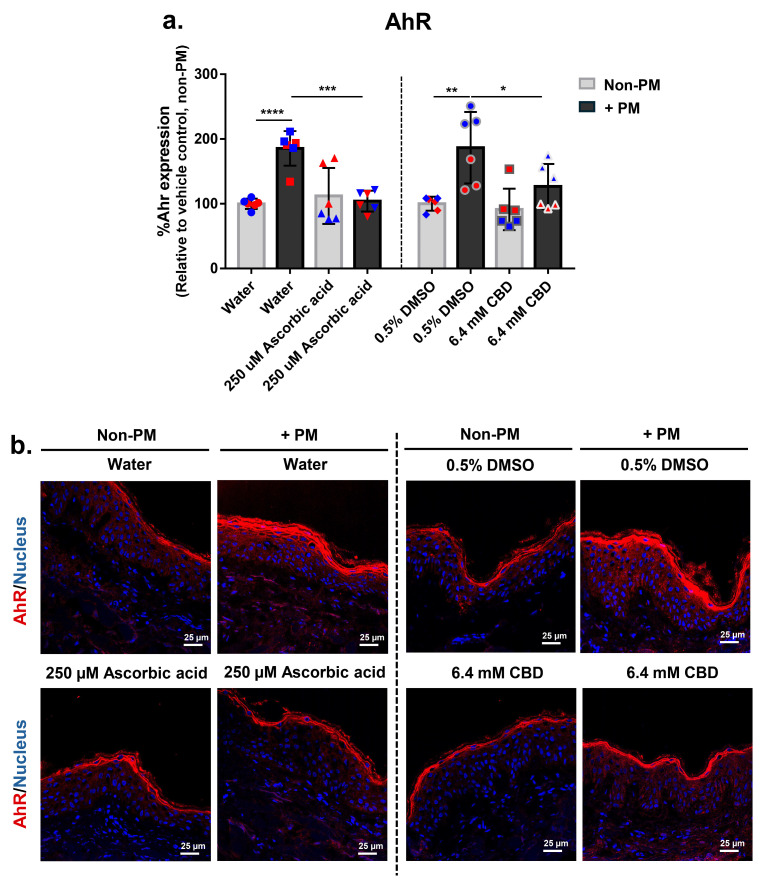
CBD attenuates PM-induced AhR expression in human ex vivo skin explants. Full-thickness human skin explants from two donors (ages 59 and 63 years; *n* = 6 tissues per group; 3 tissues per donor) were treated for 48 h with 6.4 mM CBD, 250 µM ascorbic acid, water, or 0.5% DMSO, with or without topical PM exposure (0.54 mg/cm^2^). (**a**) Quantification of AhR protein expression in skin tissues by immunofluorescence staining, expressed as % AhR expression relative to the non-PM vehicle control; (**b**) Representative immunofluorescence images from donor 2 showing AhR (red) and nuclei (blue). Scale bars = 25 µm. Each dot in (**a**) represents an individual explant; dot colors indicate donor origin (Red = donor 1 [59 years], Blue = donor 2 [63 years]). Gray bars represent non-PM groups; black bars indicate PM-exposed groups. Data are shown as mean ± SD. Asterisks indicate significance: * *p* < 0.05, ** *p* < 0.01, *** *p* < 0.001, **** *p* < 0.0001 (one-way ANOVA with Bonferroni’s post hoc test).

To investigate the potential modulatory effect of CBD on xenobiotic-sensing pathways, expression of AhR was assessed in human ex vivo skin explants following exposure to PM. AhR protein levels were evaluated by immunofluorescence staining and quantitative image analysis (Figure 4a,b). PM exposure significantly increased AhR expression by an average of 1.9-fold in both vehicle-treated groups (water and 0.5% DMSO), indicating activation of the AhR pathway in response to environmental stressors.

Treatment with 250 µM ascorbic acid reduced PM-induced AhR expression by 44%, while 6.4 mM CBD suppressed AhR levels by 32% under PM conditions. Neither treatment altered AhR expression under non-PM conditions, suggesting selective inhibition of inducible, but not baseline, AhR signaling (Figure 4a). Representative images of skin sections supported these findings, showing visibly reduced epidermal AhR staining in ascorbic acid- and CBD-treated, PM-exposed tissues compared to vehicle controls (Figure 4b). These findings suggest that CBD may modulate AhR expression in response to environmental stress, supporting its broader role in protecting skin against pollution-related changes.

### 3.7. CBD Restores PM-Induced Decline in Extracellular Matrix Markers PIP and Fibrillin in Human Skin Explants

**Figure 5:**
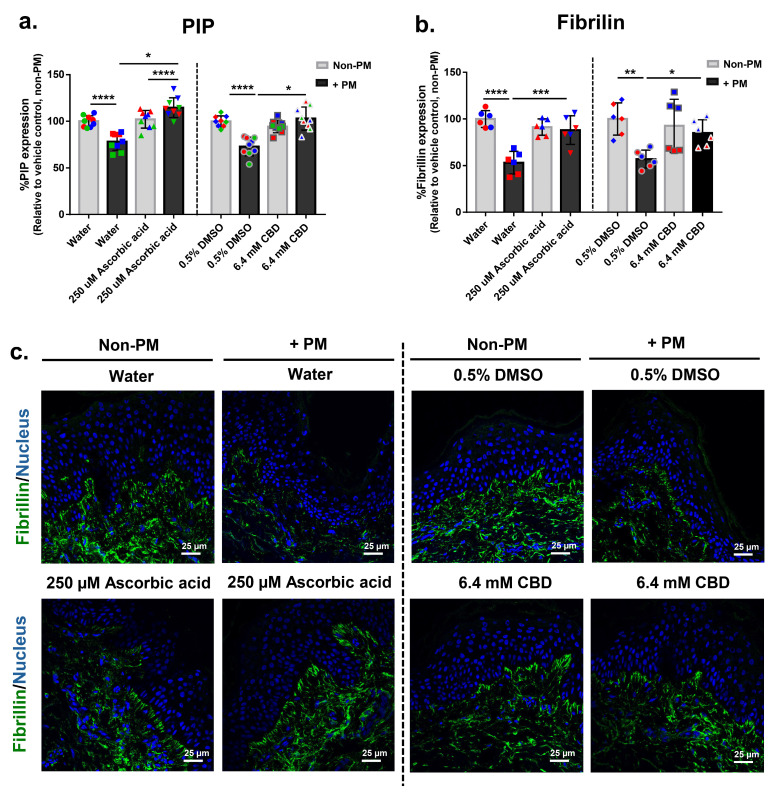
CBD restores PM-induced reductions in extracellular matrix proteins PIP and fibrillin in human ex vivo skin explants. Full-thickness human skin explants from three donors (ages 36–63 years; 3 tissues per donor) were treated for 48 h with 6.4 mM CBD, 250 µM ascorbic acid, water, or 0.5% DMSO, with or without topical PM exposure (0.54 mg/cm^2^). (**a**) PIP expression (% relative to non-PM vehicle control; *n* = 9 tissues per group; 3 tissues per donor); (**b**) Quantification of fibrillin protein expression in skin tissues by immunofluorescence staining, expressed as % Fibrillin expression relative to the non-PM vehicle control (*n* = 6 tissues per group; 3 tissues per donor). (**c**) Representative immunofluorescence images from donor 1 showing fibrillin (green) and nuclei (blue). Scale bars = 25 µm. Each dot represents an individual explant; dot colors indicate donor origin (Red = donor 1 [59 years], Blue = donor 2 [63 years], Green = donor 3 [36 years]). Gray bars represent non-PM groups; black bars indicate PM-exposed groups. Data are shown as mean ± SD. Asterisks: * *p* < 0.05, ** *p* < 0.01, *** *p* < 0.001, **** *p* < 0.0001 (one-way ANOVA with Bonferroni’s post hoc test).

To examine the effects of CBD on ECM integrity, the expression of PIP and fibrillin was analyzed in full-thickness human skin explants following PM exposure. PM treatment significantly reduced PIP expression to an average of 0.8-fold relative to the non-PM control in vehicle-treated groups (water and DMSO), indicating impaired collagen synthesis under pollutant stress (Figure 5a). Ascorbic acid reversed this decline, increasing PIP levels by 47% relative to the PM-exposed water control and exceeding its own non-PM baseline, without affecting PIP in non-stressed tissues. Similarly, CBD restored PIP expression by 41% under PM conditions, bringing levels close to the non-PM baseline, while showing no significant change in the absence of PM (Figure 5a).

Fibrillin expression followed a comparable pattern. PM exposure led to an average reduction to 0.6-fold relative to the non-PM control in both vehicle control groups, consistent with ECM degradation. Treatment with ascorbic acid elevated fibrillin expression by 66% in PM-exposed tissues, while CBD treatment restored fibrillin levels by 49%, with no effect observed in non-PM conditions (Figure 5b). Immunofluorescence staining corroborated the quantitative data, showing visible restoration of fibrillin in CBD- and ascorbic acid-treated tissues under PM stress (Figure 5c). These findings indicate that CBD counteracts pollutant-induced ECM breakdown, preserving structural components critical to skin integrity under environmental stress.

### 3.8. CBD Restores PM-Induced Loss of Filaggrin Expression in Human Skin Explants

**Figure 6:**
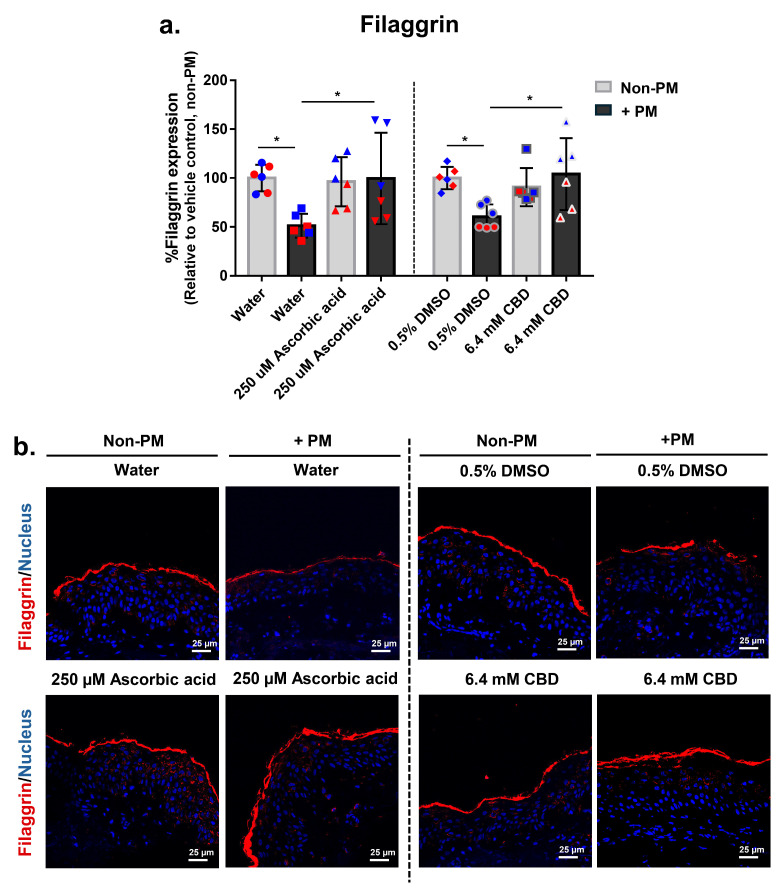
CBD restores PM-induced loss of filaggrin expression in human ex vivo skin explants. Full-thickness human skin explants from two donors (ages 59 and 63 years; *n* = 6 tissues per group; 3 tissues per donor) were treated for 48 h with 6.4 mM CBD, 250 µM ascorbic acid, water, or 0.5% DMSO, with or without topical PM exposure (0.54 mg/cm^2^). (**a**) Quantification of filaggrin protein expression in skin tissues by immunofluorescence staining, expressed as % Filaggrin expression relative to the non-PM vehicle control; (**b**) Representative immunofluorescence images from donor 2 showing filaggrin (red) and nuclei (blue). Scale bars = 25 µm. Each dot in (**a**) represents an individual explant; dot colors indicate donor origin (Red = donor 1 [59 years], Blue = donor 2 [63 years]). Gray bars represent non-PM groups; black bars indicate PM-exposed groups. Data are shown as mean ± SD. Asterisks indicate significance: * *p* < 0.05 (one-way ANOVA with Bonferroni’s post hoc test).

To evaluate the effect of CBD on skin barrier function, filaggrin expression was assessed in full-thickness human skin explants exposed to PM. Quantitative immunofluorescence analysis showed that PM exposure significantly reduced filaggrin levels to an average of 0.6-fold relative to the non-PM control in both vehicle-treated groups (water and 0.5% DMSO), consistent with barrier disruption and impaired epidermal differentiation (Figure 6a,b).

Treatment with 250 µM ascorbic acid increased filaggrin expression by 94% under PM conditions compared to the PM-exposed water control, while having no significant effect in non-PM tissues. Similarly, 6.4 mM CBD restored filaggrin expression by 63% in PM-exposed explants but did not alter baseline expression in the absence of PM exposure (Figure 6a). Immunofluorescence staining confirmed these findings, showing enhanced epidermal filaggrin localization in PM-exposed tissues treated with either ascorbic acid or CBD (Figure 6b). These results demonstrate that CBD mitigates pollution-induced loss of filaggrin, supporting its potential to preserve skin barrier integrity under environmental stress.

Although baseline levels differed between donors, the pattern of PM-induced changes and CBD-mediated protection was consistent across all donors included in each assay, as illustrated by the color-coded data points in the figures. A consolidated overview of PM-induced changes and CBD-mediated modulation across all biomarkers, including inflammation, oxidative stress, AhR signaling, ECM integrity, and barrier function, is provided in Table S1 to facilitate rapid comparison of treatment effects.

## 4. Discussion

Airborne PM is a major contributor to extrinsic skin aging, inflammation, and barrier dysfunction. It activates xenobiotic sensors such as AhR, promotes ROS generation, and disrupts structural proteins including collagen and filaggrin [8,36,37]. Using a full-thickness human ex vivo skin model, this study demonstrates that CBD, administered via culture medium to simulate systemic exposure, significantly mitigates PM-induced skin damage. CBD suppressed key inflammatory markers and oxidative stress, modulated AhR signaling, and restored extracellular matrix and barrier integrity, all without compromising tissue viability, supporting its potential as a protective agent against environmental stress.

The PM dose (0.54 mg/cm^2^) reliably induced hallmark features of pollution-related skin pathology, including elevated IL-6, MMP-1, COX-2, ROS, and 8-OHdG, as well as increased AhR expression and downregulation of PIP, fibrillin, and filaggrin. These effects are consistent with prior reports linking PM exposure to AhR activation, oxidative DNA damage, inflammatory signaling, and structural tissue degradation [33,38,39,40]. CBD exerted a robust anti-inflammatory response, significantly reducing IL-6, MMP-1, and COX-2 levels under PM exposure (Figure 1b,c and Figure 2a,b), in line with previous findings showing that CBD decreased IL-6 in HaCaT keratinocytes exposed to PM [22]. While CBD’s anti-inflammatory effects in monolayer keratinocyte models are well established [26,41,42,43], our results extend these observations to a complex, viable human skin environment. Notably, MMP-1 suppression occurred under both basal and stressed conditions, suggesting that CBD may exert matrix-protective effects partly independent of inflammation, potentially via AP-1/MAPK modulation as previously proposed [22,44].

A central finding of this study is the significant reduction of oxidative stress markers by CBD. ROS levels decreased by 29%, and oxidative DNA damage (8-OHdG) by 20% following CBD treatment under PM conditions (Figure 3a,b), while no effect was observed in non-stressed skin. This contrasts with PM-exposed HaCaT keratinocytes, in which CBD did not markedly inhibit ROS generation and showed only modest activity in cell-free antioxidant assays [22]. Several factors may explain this difference. First, our full-thickness explants contain multiple interacting cell types and an intact extracellular matrix, providing a more integrated redox network than a keratinocyte monolayer, so CBD may lower oxidative burden indirectly by modulating stress-responsive pathways, including activation of Nrf2-driven antioxidant responses and suppression of pro-oxidant enzymes such as NOX and iNOS [45]. Second, PM and CBD exposure conditions differ between the models (ex vivo versus 2D monolayer, exposure duration, and readout compartment), so the 48 h response in our explants likely captures both primary ROS generation and downstream redox adaptation. The absence of CBD effects on ROS in non-stressed skin further supports a stress-inducible redox modulator, which is advantageous in dermatologic applications where preserving physiological redox balance is essential.

Importantly, CBD also reduced PM-induced upregulation of AhR expression, a key sensor involved in pollutant-driven inflammation and oxidative stress (Figure 4a,b). Previous studies reported that CBD activates AhR in normal human keratinocytes and 3D epidermal models under non-stressed conditions [30]. In our full-thickness ex vivo model, however, CBD did not change AhR expression in non-PM groups but selectively attenuated its induction under PM challenge. These differences likely reflect the use of adult full-thickness skin with higher basal AhR tone and different readouts of AhR signaling (total protein expression in our study versus nuclear translocation and downstream targets). Together, our findings support the idea that, in pollution-stressed human skin, CBD acts to restrain excessive AhR upregulation without perturbing basal expression, thereby helping to maintain balanced AhR signaling under environmental stress [46].

These protective effects extended to both extracellular matrix and epidermal barrier components. CBD restored PIP and fibrillin expression, key markers of collagen synthesis and elastic fiber integrity (Figure 5a–c), and reversed PM-induced loss of filaggrin, a critical protein for epidermal differentiation and moisture retention (Figure 6a,b), while having little or no effect on these markers under non-PM conditions. This pattern is consistent with a recent ex vivo anti-aging model in which CBD did not increase collagen in non-stressed human skin explants [22], and with reports that CBD promotes keratinocyte differentiation, upregulates filaggrin, and supports matrix remodeling under oxidative stress [30,47,48]. Together, these findings suggest that CBD primarily normalizes pollution-induced ECM and barrier disruption rather than stimulating collagen synthesis in unstressed skin, underscoring its dual role in preserving structural and barrier integrity in environmentally stressed skin.

In our main multi-donor experiment, three female donors aged 36–63 years contributed full-thickness abdominal skin explants. As expected for human skin, baseline levels and the magnitude of PM- and CBD-induced responses varied between donors. However, PM consistently induced the same directional changes, and CBD consistently attenuated these changes in all donors for both biochemical and immunofluorescence outcomes. This design therefore incorporates inter-individual, including age-related, variability while still demonstrating a coherent protective profile of CBD in human ex vivo skin.

The concentrations used in this study reflect established practices in ex vivo research. Although the PM dose (0.54 mg/cm^2^) exceeds typical ambient exposure levels, it is commonly applied to model cumulative or high-dose scenarios in controlled systems lacking active detoxification pathways [33,49,50,51]. Likewise, the 6.4 mM CBD concentration used here (≈2.0 mg/mL in culture medium) surpasses the low micromolar plasma levels typically observed in vivo following oral and transdermal administration, due to extensive first-pass metabolism, protein binding, and limited bioavailability [52,53], but is appropriate for a static, non-perfused explant system in which blood flow, metabolic clearance, and physiological dilution are absent [16]. In this context, CBD administration via the culture medium is used to mimic systemic delivery and to establish a stable exposure gradient across the tissue, analogous to actives reaching the skin via the dermal microvasculature in vivo [31,32]. Similar supraphysiologic test concentrations have been used in other medium-based ex vivo human skin models to interrogate mechanistic responses [31,32]. Our findings should therefore be interpreted as demonstrating the biological potential of CBD to counteract pollution-induced damage under systemic-mimicking conditions, rather than as defining clinically achievable systemic doses.

In addition to clinical exposure levels, CBD concentrations also vary widely across experimental skin models. A previous study used 10 µM CBD in a PM-exposed HaCaT keratinocyte monolayer, but a much higher nominal dose of 0.5% (*w*/*v*) CBD (≈16 mM) was used for topical application in ex vivo human skin [22]. This difference reflects both model geometry and exposure route: in 2D cultures, keratinocytes are uniformly bathed in medium, so low micromolar concentrations are sufficient, whereas full-thickness explants are three-dimensional tissues with dense extracellular matrix and no vascular perfusion, requiring diffusion through collagen-rich dermis and binding to tissue components [54]. In our system, CBD is delivered from the medium side without a stratum corneum barrier, while topical application must first overcome the stratum corneum, a strong diffusion barrier that necessitates higher nominal doses to reach viable layers [55]. Thus, our 6.4 mM mechanistic dose, delivered via the culture medium, reasonably falls between the 10 µM used in 2D keratinocytes and the 0.5% (~16 mM) topical dose used in ex vivo skin, and was selected to ensure adequate tissue exposure in this static explant model.

The full-thickness human skin explant model preserves native epidermal–dermal architecture, resident immune cells, and extracellular matrix components, making it a valuable platform for translational skin research [31,32,33]. Nevertheless, several limitations should be considered when interpreting the findings. First, this model is a static, non-perfused system and therefore does not reproduce systemic physiology, including vascular perfusion, metabolism, immune-cell trafficking, or interactions with the microbiome [16,56,57,58]. Second, the supraphysiologic CBD concentration used here was deliberately selected as a mechanistic exposure to ensure sufficient tissue penetration in a non-perfused system, rather than to represent clinically achievable systemic levels. Third, the donor cohort consisted of adult female abdominoplasty patients (36–63 years), which may limit direct extrapolation to other age groups, sexes, and anatomical sites. Finally, the experimental design focused on an acute 48 h exposure to PM and thus does not capture potential long-term or cumulative effects of pollution or CBD treatment. Future studies using perfused or microfluidic skin platforms, dermal penetration and pharmacokinetic analyses for different delivery routes (oral, nutricosmetic, and topical), larger and more diverse donor populations, and in vivo nutricosmetic or clinical trials will be essential to confirm and extend these mechanistic observations and to define their relevance to real-world human exposure scenarios.

From a safety perspective, pure CBD, unlike THC, is generally regarded as a non-intoxicating cannabinoid with no evidence of abuse or dependence potential in humans [21,22]. In the cosmetic context, the Scientific Committee on Consumer Safety (SCCS) has considered CBD safe for use in dermal cosmetic products at concentrations up to 0.19% (*w*/*w*) under current exposure assumptions [59], and additional in vitro and ex vivo skin studies support its dermal safety at low-to-moderate concentrations relevant for cosmetic use [22]. Although our work did not apply CBD topically or measure dermal permeation and therefore cannot directly inform product-use levels, these regulatory and preclinical data indicate that, within presently evaluated exposure ranges, CBD has a favorable safety profile that complements the mechanistic anti-pollution protection observed in our ex vivo model.

## 5. Conclusions

This study provides compelling evidence that CBD protects against pollution-induced skin damage by modulating inflammatory, oxidative, and xenobiotic stress pathways. In addition to biochemical modulation, CBD restored extracellular matrix and barrier integrity, supporting its potential as a functional ingredient for promoting skin health in urban environments. While these findings are based on a static ex vivo model and a mechanistic CBD exposure, they provide a translational framework and biomarker set that support further investigation of CBD in systemic (nutricosmetic) and topical applications.

## Data Availability

The raw data supporting the conclusions of this article will be made available by the authors on request. This restriction is due to the dataset being part of an ongoing research project, where further analysis and follow-up studies are planned. However, data may be shared upon reasonable requests for academic or research purposes.

## Supplementary Materials

The following supporting information can be downloaded at: https://www.mdpi.com/article/10.3390/biom16010010/s1, Figure S1. PM dose optimization in human ex vivo skin explants; Figure S2. CBD concentration selection study in PM-exposed human skin explants: evaluation of anti-inflammatory and tissue viability effects; Figure S3. CBD concentration selection study in PM-exposed human skin explants: effects on collagen type I expression; Table S1. Summary of PM-induced alterations and CBD-mediated modulation of key biomarkers in human ex vivo full-thickness skin explants.
