# Supplementary material for: Cannabidiol Mitigates Pollution-Induced Inflammatory, Oxidative, and Barrier Damage in Ex Vivo Human Skin
Source: Biomolecules. 2025 Dec 20;16(1):10. doi: 10.3390/biom16010010 (PMC12838751; doi:10.3390/biom16010010)
Supplement: Supplementary file 1 [file biomolecules-16-00010-s001.zip › Supplementary data.pdf]

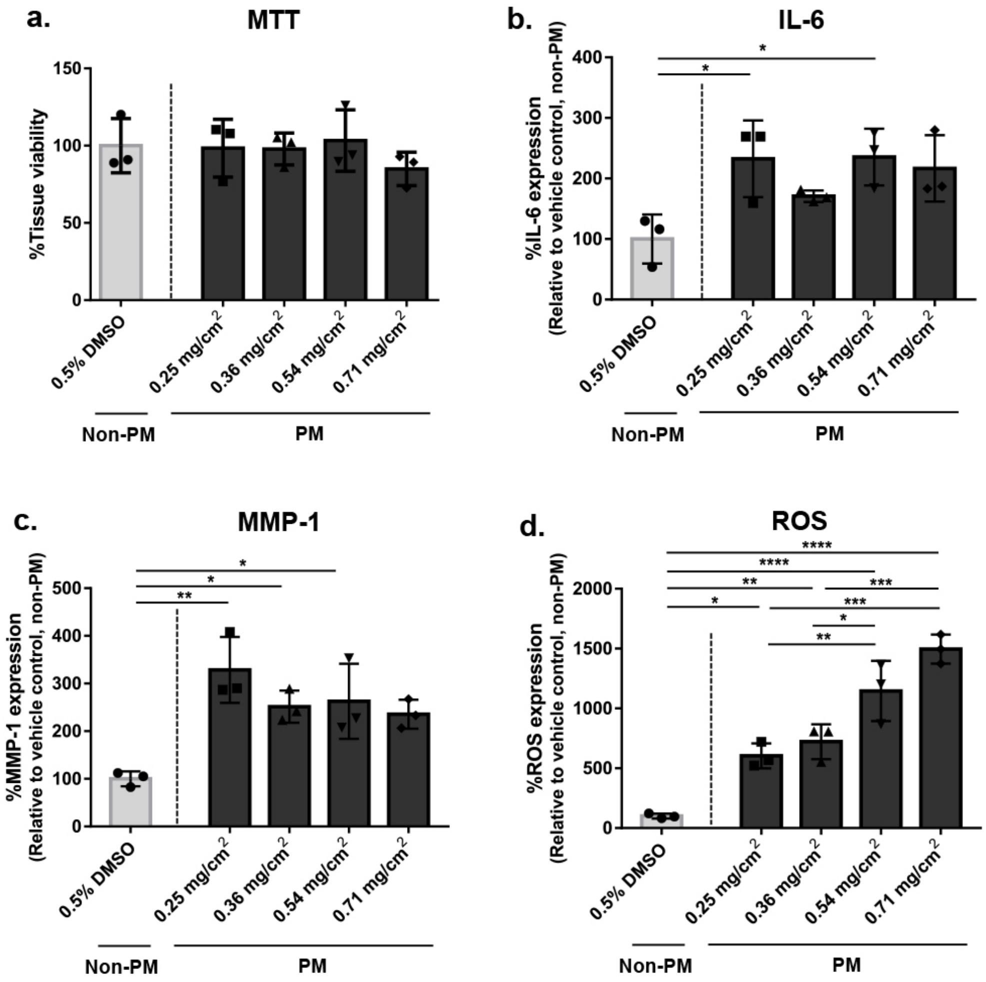

Figure S1. PM dose optimization in human *ex vivo* skin explants. Full-thickness human skin explants from one donor (53 years old) were topically exposed to increasing doses of PM (0.25, 0.36, 0.54, and 0.71 mg/cm²) for 24 h to determine an effective concentration for pollution-induced skin damage. Explants treated with 0.5% DMSO served as non-PM vehicle controls. **(a)** Tissue viability assessed by MTT assay and expressed as % relative to non-PM control; **(b)** IL-6 expression; **(c)** MMP-1 expression; **(d)** ROS generation — all quantified in culture medium and expressed as % relative to non-PM vehicle control. Data represent mean ± SD (n = 3 tissues per group). Statistical significance was determined by one-way ANOVA followed by Dunnett's post hoc test for tissue viability and Tukey's multiple comparisons test for all other parameters. *p < 0.05, **p < 0.01, ***p < 0.001, ****p < 0.0001.

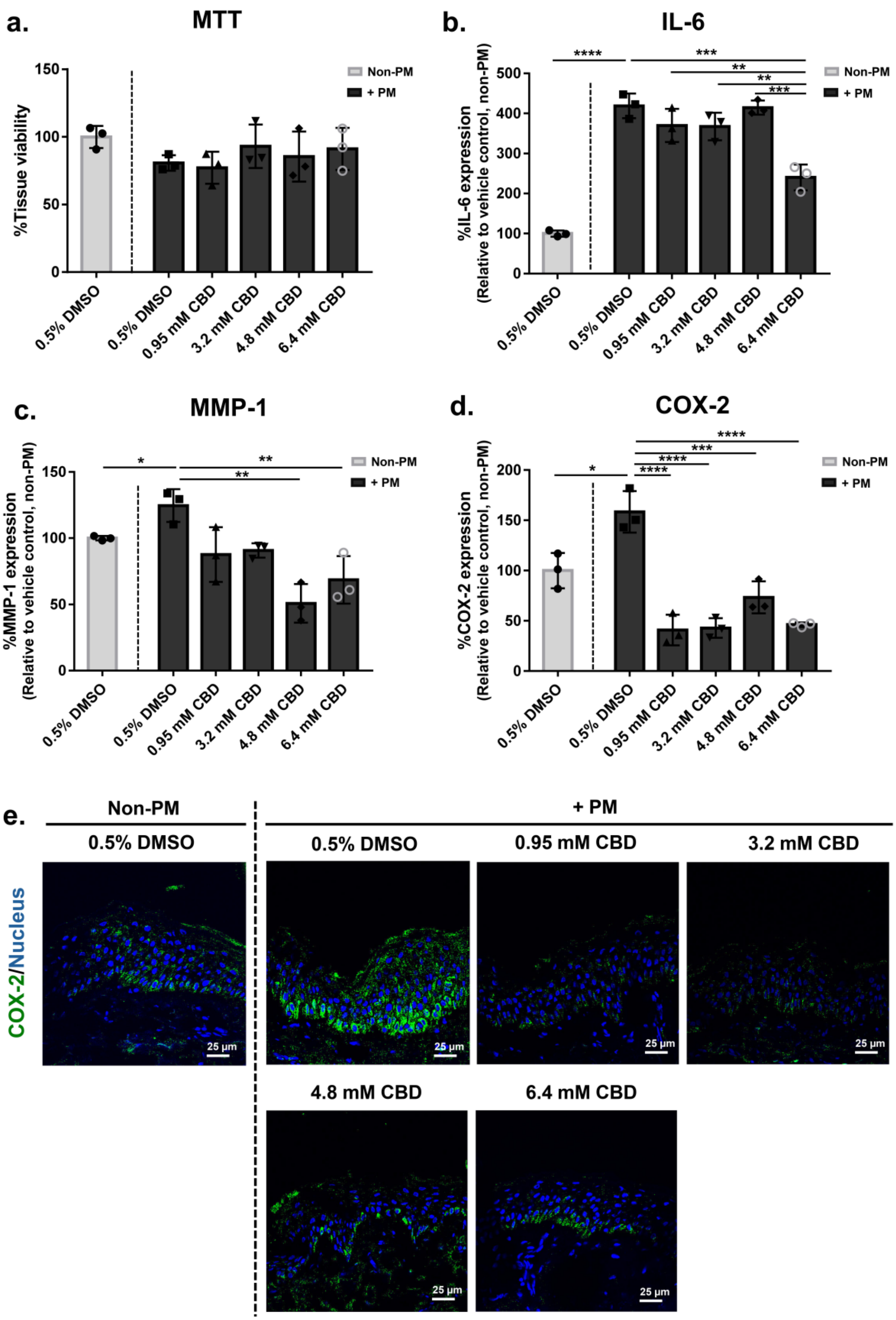
a.
MTT
%Tissue viability
150
100
50
0
Non-PM
+ PM
0.5% DMSO
0.5% DMSO
0.95 mM CBD
3.2 mM CBD
4.8 mM CBD
6.4 mM CBD
b.
IL-6
%IL-6 expression
(Relative to vehicle control, non-PM)
500
400
300
200
100
0
****
***
**
**
***
Non-PM
+ PM
0.5% DMSO
0.5% DMSO
0.95 mM CBD
3.2 mM CBD
4.8 mM CBD
6.4 mM CBD
c.
MMP-1
%MMP-1 expression
(Relative to vehicle control, non-PM)
150
100
50
0
*
**
**
Non-PM
+ PM
0.5% DMSO
0.5% DMSO
0.95 mM CBD
3.2 mM CBD
4.8 mM CBD
6.4 mM CBD
d.
COX-2
%COX-2 expression
(Relative to vehicle control, non-PM)
200
150
100
50
0
*
****
***
****
****
Non-PM
+ PM
0.5% DMSO
0.5% DMSO
0.95 mM CBD
3.2 mM CBD
4.8 mM CBD
6.4 mM CBD
e.
Non-PM
+ PM
0.5% DMSO
0.5% DMSO
0.95 mM CBD
3.2 mM CBD
COX-2/Nucleus
25 µm
4.8 mM CBD
6.4 mM CBD

Figure S2. CBD concentration selection study in PM-exposed human skin explants: evaluation of anti-inflammatory and tissue viability effects. Full-thickness human skin explants from a single donor (40 years old) were treated with increasing concentrations of CBD (0.95, 3.2, 4.8, and 6.4 mM) via the culture medium for 48 h, in the presence or absence of 0.54 mg/cm^2 topical PM exposure. **(a)** Tissue viability assessed by MTT assay; **(b)** IL-6 expression; **(c)** MMP-1 expression; **(d)** COX-2 expression —all reported as % relative to non-PM vehicle control (0.5% DMSO); **(e)** Representative immunofluorescence images of COX-2 expression (green) and nuclei (blue). Scale bars = 25 µm. Data are presented as mean ± SD (n = 3 tissues per group). Statistical analysis: one-way ANOVA followed by Dunnett's post hoc test for tissue viability and Tukey's post hoc test for cytokine/protein expression. *$p < 0.05$, **$p < 0.01$, ***$p < 0.001$, ****$p < 0.0001$.

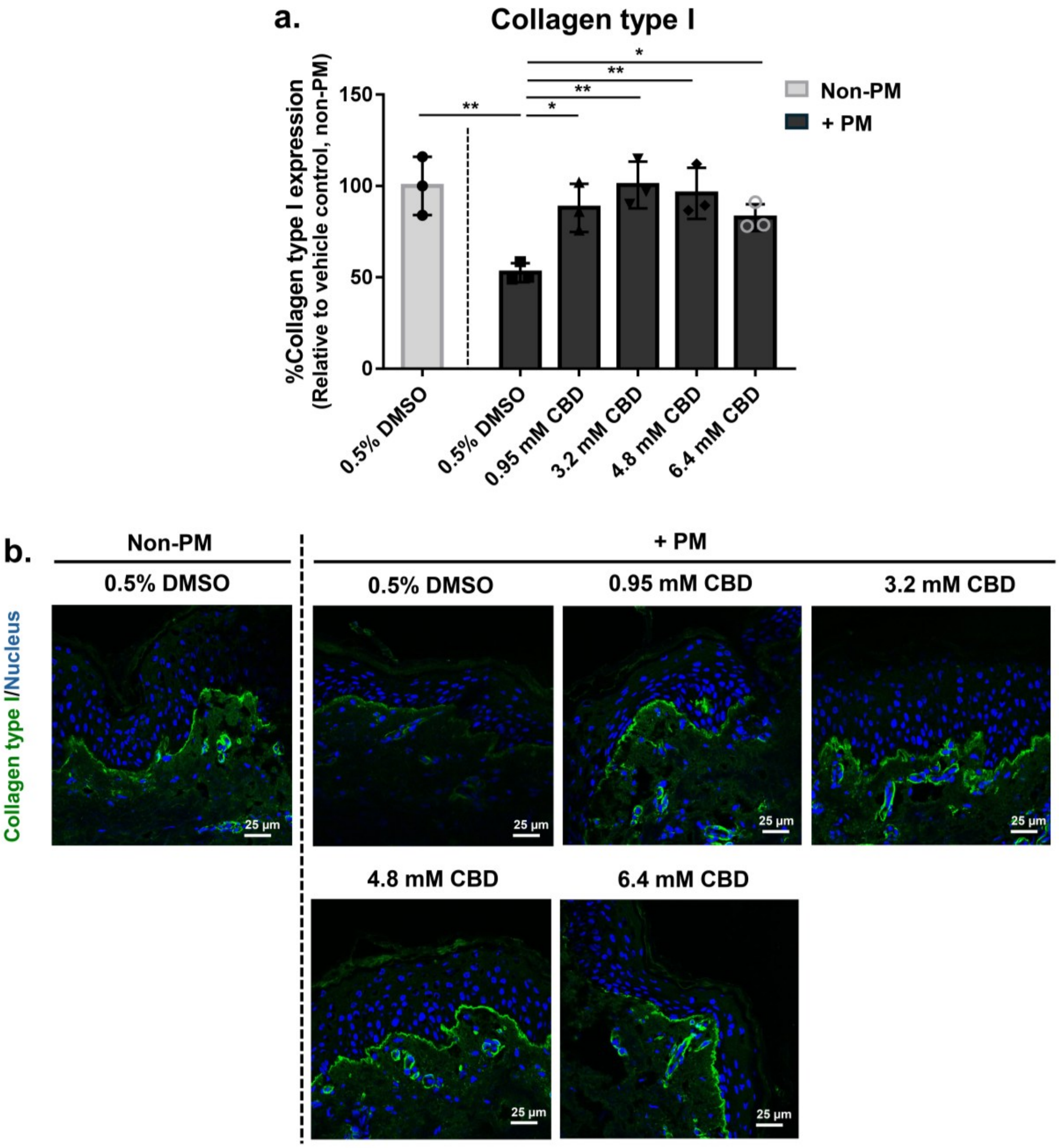

Figure S3. CBD concentration selection study in PM-exposed human skin explants: effects on collagen type I expression. Full-thickness human skin explants from a single donor (40 years old) were treated with increasing concentrations of CBD (0.95, 3.2, 4.8, and 6.4 mM) via the culture medium for 48 h, in the presence or absence of 0.54 mg/cm² topical PM exposure. **(a)** Quantification of collagen type I expression (% relative to non-PM vehicle control); **(b)** Representative immunofluorescence images showing collagen

type I (green) and nuclei (blue). Scale bars = 25 μm. Data are shown as mean ± SD ($n = 3$ tissues per group). Statistical analysis: one-way ANOVA followed by Tukey's multiple comparisons test. *$p < 0.05$, **$p < 0.01$.

Table S1. Summary of PM-induced alterations and CBD-mediated modulation of key biomarkers in human *ex vivo* full-thickness skin explants.

Biomarker	PM effect[1] (Non-PM vs. PM)	CBD effect in baseline (DMSO vs CBD, non-PM)	CBD effect under PM (DMSO vs CBD, PM)
**Inflammation**			
IL-6	↑ 3.4-fold	ns	↓ 63% ***
MMP-1	↑ 2.0-fold	↓ 60% **	↓ 61% ****
COX-2	↑ 1.7-fold	ns	↓ 61% ***
**Oxidative stress**			
ROS	↑ 1.9-fold	ns	↓ 29% *
8-OHdG	↑ 1.2-fold	ns	↓ 20% ***
**Xenobiotic-sensing**			
AhR	↑ 1.9-fold	ns	↓ 32% *
**Extracellular matrix**			
PIP	↓ 0.8-fold	ns	↑ 41% *
Fibrillin	↓ 0.6-fold	ns	↑ 49% *
**Skin barrier**			
Filaggrin	↓ 0.6-fold	ns	↑ 63% *

[1] Values in the "PM effect" column represent the mean fold-change between non-PM and PM conditions, calculated from all treatment groups in which PM exposure caused a statistically significant change. ns = not significant. Asterisks: *$p < 0.05$, **$p < 0.01$, ***$p < 0.001$, ****$p < 0.0001$ (one-way ANOVA with Bonferroni's post hoc test).
